# The representation of visual depth perception based on the plenoptic function in the retina and its neural computation in visual cortex V1

**DOI:** 10.1186/1471-2202-15-50

**Published:** 2014-04-23

**Authors:** Zhao Songnian, Zou Qi, Liu Chang, Liu Xuemin, Sun Shousi, Qiu Jun

**Affiliations:** 1 LAPC, Institute of Atmospheric Physics, Chinese Academy of Sciences, Beijing 100029, China; 2 Computer Science and Technology, Beijing Jiaotong University, Beijing 100044, China; 3 Beijing Information Science and Technology University, Beijing 100101, China

**Keywords:** Plenoptic function, Visual perception, Three-dimensional scene, Vanishing point, Retina, Primary visual cortex, Neural computation, Affine transformation

## Abstract

**Background:**

How it is possible to “faithfully” represent a three-dimensional stereoscopic scene using Cartesian coordinates on a plane, and how three-dimensional perceptions differ between an actual scene and an image of the same scene are questions that have not yet been explored in depth. They seem like commonplace phenomena, but in fact, they are important and difficult issues for visual information processing, neural computation, physics, psychology, cognitive psychology, and neuroscience.

**Results:**

The results of this study show that the use of plenoptic (or all-optical) functions and their dual plane parameterizations can not only explain the nature of information processing from the retina to the primary visual cortex and, in particular, the characteristics of the visual pathway’s optical system and its affine transformation, but they can also clarify the reason why the vanishing point and line exist in a visual image. In addition, they can better explain the reasons why a three-dimensional Cartesian coordinate system can be introduced into the two-dimensional plane to express a real three-dimensional scene.

**Conclusions:**

1. We introduce two different mathematical expressions of the plenoptic functions, *P*_*w*_ and *P*_*v*_ that can describe the objective world. We also analyze the differences between these two functions when describing visual depth perception, that is, the difference between how these two functions obtain the depth information of an external scene.

2. The main results include a basic method for introducing a three-dimensional Cartesian coordinate system into a two-dimensional plane to express the depth of a scene, its constraints, and algorithmic implementation. In particular, we include a method to separate the plenoptic function and proceed with the corresponding transformation in the retina and visual cortex.

3. We propose that size constancy, the vanishing point, and vanishing line form the basis of visual perception of the outside world, and that the introduction of a three-dimensional Cartesian coordinate system into a two dimensional plane reveals a corresponding mapping between a retinal image and the vanishing point and line.

## Background

How a three-dimensional scene can be “faithfully” expressed in a (two-dimensional) plane (e.g., TV), that is to say, how it can be “faithfully” represented using a planar Cartesian coordinate system, and what the differences are between the stereoscopic perception of an actual scene and its two-dimensional image are important issues in visual information processing research, neural computation, psychophysics, and neuroscience.

At the cellular level, previous studies have shown that in the V1 cortex, only complex cells are able to respond to absolute parallax [1]. In the V2 cortex, there are some cortical neurons that respond to relative parallax [2] and parallax-sensitive neurons can be described by specific and generalized energy models [3,4]. Studies have been carried out both in the ventral and dorsal streams of the visual cortex, mainly to detect neurons that can respond to depth perception through specific signal stimuli. The binocular visual system is able to perceive depth information using binocular disparity, and one of the founders of the computational theory of vision, Marr, proposed a classic reconstruction algorithm for three-dimensional images [5]. Julesz’s experiments on random dot stereograms (RDSs) led to a psychophysical study on the binocular disparity that forms stereoscopic vision. Its purpose was to show how the human brain deals with depth information [6,7]. In other words, the task was to explore how human vision extracted stereoscopic information from a visual scene contained in a Cartesian coordinate system and depicted on a two-dimensional imaging plane.

A three-dimensional scene “faithfully” represented in a plane seems to be commonplace phenomenon, yet the mechanism for this has never been explored. It is, however, a basic theoretical problem and is worthy of study in depth, not only because it concerns the geometric and physical properties of planes and space and is closely related to the three-dimensional perception of human vision, but also because it is closely related to the problem of stereoscopic perception in computer vision, robotics navigation, and visual cognitive psychology.

In fact, there are many similar phenomena, such as optical illusions generated using optics, geometry, physiology, psychology, and other means. Optical illusions are largely due to the uncertainty caused by the bimodal graphics in a two-dimensional plane and uncertainty during visual information processing in the brain. The illusions, such as bimodal images for instance (vase and face, girl and grandmother, Escher’s “waterfall” picture, and so on) and Additional file 1 disappear when the images are placed in a real three-dimensional space. Additional files 2, 3, 4, and 5 show the lifelike effect of three-dimensional perception, can be more intuitively reflect the meaning f this article.

Marr pointed out that the essence of visual information processing is to discover what and where objects are in space [5]. F. Crick also stated that visual information processing is a construction process [8]. In their book *Seeing*, Frisby and Stone defined how “seeing” is a particularly difficult task. They analyzed research from computational vision, psychophysics, neurobiology, neuroanatomy, brain imaging, modeling methods, image statistics, multiple representations, active vision, Bayesian theory, and the philosophy of visual information processing. The understanding of “seeing” among these fields is not the same, each focuses on different aspects of “seeing”, and each has their own understanding of the “the essence of seeing” (for details, see Chapter 23 of [9]).

As is known, any point in space can be represented by a Cartesian coordinate system (*x*, *y*, *z*) at a certain moment, *t*, and an object at this point can be expressed using light intensity *V*_*x*_, *V*_*y*_, *V*_*z*_ and color-related wavelength λ. In this way, one can define a function *P*_*w*_, *P*_w_ = *P*_w_(*x*, *y*, *z*; *λ*, *V*_*x*_, *V*_*y*_, *V*_*z*_; *t*), that completely represents an object, and is also a good description of the objective external world. When human vision processes an object, the optical axis of the eyeball is consistent with the *z* axis (the depth axis), such that the visual imaging plane is perpendicular to the optical axis. This reduces one variable from the function, *P*_*w*_, and leaves only seven variables that form the plenoptic function proposed by Adelson and Bergen in the study of human primary visual information processing [10].

The intensity of each ray can be described as a function of the spatial viewing angle; that is, the wavelength, time, and light intensity of the observation position (the expression is *P*_v_ = *P*_v_(*θ*, *ϕ*, *λ*, *t*, *V*_*ox*_, *V*_*oy*_, *V*_*oz*_) in spherical coordinates and *P*_v_ = *P*_v_(*x*, *y*; *λ*, *V*_*ox*_, *V*_*oy*_, *V*_*oz*_; *t*) in Cartesian coordinates) captures all that the human eye or optical device may “see”, including ambient light. Therefore, the plenoptic function and full holographic representation of the visible world are equivalent. As for the different definitions of the plenoptic function and its mathematical expression, we will discuss this in some detail in the discussion [10,11].

We should note that the plenoptic function not only reveals how humans “see” the external world, but also intuitively and concisely describes the information processing that occurs between the retina and the primary visual cortex. Marr pointed out that the true nature of information processing in “seeing” is to discover where and what is in space. “Where” in space can be located by a Cartesian rectangular coordinate system (i.e., x, y, and z). “What” is in this position may be perceived through the emitted or reflected structure of the light ray from the “object” to the viewer’s eyes, These correspond to the intensity *V*_*x*_, *V*_*y*_, *V*_*z*_ and wavelength λ of light at that location that carry information about the contour, shape, and color of the object. Thus, it can be seen that the plenoptic function is a good description of the external world. When Adelson and Bergen proposed the plenoptic function, their intentions were to solve the problem related to the corresponding points in computer vision. It was not expected that the study would promote the birth and development of the new discipline of computational photography [12-16]. To adapt to the needs of different disciplines, there are two basic formulae for the plenoptic function, one describes an object *P*_*W*_ = *P*(*x*, *y*, *z*; *V*_*x*_, *V*_*y*_, *V*_*z*_, *λ*, *t*) and the other describes the viewer’s perception of the object. In such a case, the optical axis (or possibly the visual axis) of human vision and the coordinate axis z are consistent, thereby eliminating the need for coordinate axis z, namely: *P*_v_ = *P*(*x*, *y*; *V*_*x*_, *V*_*y*_, *V*_*z*_, *λ*, *t*). “Seeing” is the association between the observer and the object, where the coordinates of an observer’s position are x, y, and z, and the light intensities that an object emits or reflects to the observer’s eye are *V*_*ox*_, *V*_*oy*_, *V*_*oz*_, representing the light intensity information of the object itself. The intensity of light is related to the number of excited photo-sensitive cells in the retina and their activity levels. As long as the angles of the incident light *θ* and *φ* are recorded, the plenoptic function can be simplified as *P*_v_ = *P*(*x*, *y*; *θ*, *φ*, *λ*, *t*) such that a dual-plane (*x, y*) and (*θ*, *φ*) parameterization becomes possible. This parameterization is used in this paper and is important for processing the visual information of an image to reveal its deep meaning.

**Figure 1:**
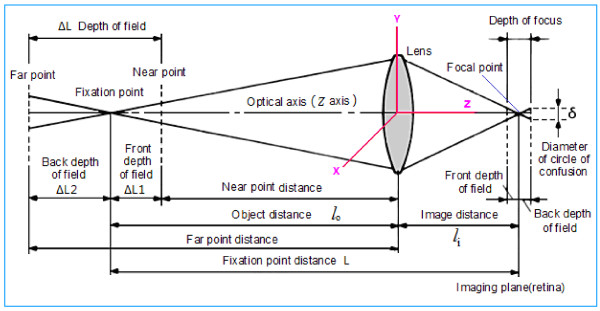
**Schematic diagram of depth of field and depth of focus [**[17]**].**

An interesting and important question concerns the difference between the functions *P*_w_ and *P*_v_. It is generally considered that *P*_w_ differs from *P*_v_ in the number of dimensions; i.e., the coordinates are reduced from (*x*, *y*, *z*) to (*x*, *y*). However, in practice, when the visual system perceives an external scene, the optical axis (or visual axis) is consistent with the *z* axis. The imaging plane is perpendicular to the optical axis (i.e., the *z* axis) and this is an inherent characteristic of the optical imaging system of vision. At a certain distance in front of and behind the focal plane of a visual image (the retina), the visual system is able to form a clear visual image. The diameter δ of this region (circle of confusion) is very small (0.005 mm) and gives us the depth of focus (Figure 1) [17]. According to the conjugate relationship between the image and object points of the viewed object, there is a similar situation. When a light spot is formed at a certain small distance before and after the object, the depth of field is formed jointly by the near and far points. The human visual system perceives the depth of field in the surrounding world through its optical system. The ΔL depth of field of imaging on the retina is jointly determined by δ, f, F, L, ΔL1, and ΔL2, where δ is the diameter of the permissible circle, f is the focal length of the lens, F is the size of the pupil, L is the focusing distance, ΔL1 is the front depth of field, and ΔL2 is the back depth of field. Then, the formula for the depth of field ΔL can be expressed as follows:

(1) $$\mathrm{\Delta L}=\mathrm{\Delta L}1+\mathrm{\Delta L}2=\frac{2f^{2}\mathrm{F\delta}L^{2}}{f^{4}-F^{2}\delta^{2}L^{2}}$$

and the focal length *f* can be calculated by

(2) $$\frac{1}{l_{o}}+\frac{1}{l_{i}}=\frac{1}{f}\;\mathrm{or}\;f=\frac{l_{o}l_{i}}{l_{o}+l_{i}}$$

Where *l*_o_ object is distance and *l*_i_ is image distance, as shown in Figure 1. Therefore, the visual image in the retina contains information about the depth of field that is not lost when the three-dimensional objective world is represented in the two-dimensional retina of the visual system. This is mainly because the optical axis is coincident with the coordinate axis *z*; that is to say, *L* and *Z* are equivalent in formula (1) and thus they can replace each other. Therefore, formula (1) can be rewritten as follows

(3) $$\mathrm{\Delta L}\left(z\right)=\mathrm{\Delta L}1+\mathrm{\Delta L}2=\frac{2f^{2}\mathrm{F\delta}z^{2}}{f^{4}-F^{2}\delta^{2}z^{2}}$$

In formula (3), *z* is the distance of the *Z* axis, reflecting depth information. It is thus clear that the coincidence of the optical axis with the coordinate axis *Z* is a very effective constraint. It is not imposed artificially, but is determined by the optics of the visual system.

**Figure 2:**
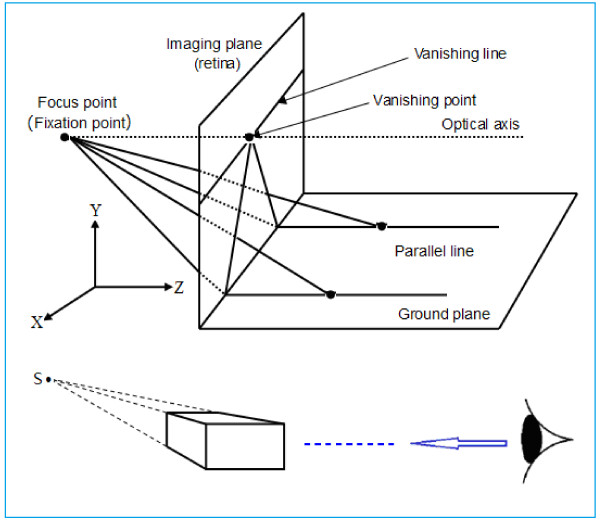
**Optics model of the affine transformation of parallel lines implemented by vision [**[18]**].** The optical axis of the vision points to a distant focus, the fixation point. Straight parallel lines converge at the focus. The focus and its vanishing line are projected on the retina or imaging plane through the vanishing line and point in the retina. The visual system then perceives a distant intersection in the scene of the external world. Figure 2 shows the Cartesian coordinate system in which the Z axis and the optical axis are consistent.

As one gazes into the distance, the depth of field may extend to infinity. One familiar phenomenon occurs when we look at a distant railway or highway and the tracks or road edges gradually converge to a single point in the distance (called the vanishing point), as shown in Figure 2. The image in the imaging plane is just a visual image on the retina. This is a result of an affine transformation of the visual optical system, but is also the objective reality or physical truth of human vision when observing the world and is a basic characteristic of visual image processing. The fact that the visual system perceives the railroad tracks converging at one point in the distance demonstrates that there is not a corresponding point in the Cartesian coordinate system. However, it is easy to solve this problem by supplementing a new coordinate point (*α*) with a homogeneous coordinate, thereby establishing the mapping relationship between the Cartesian coordinate system *R*^*n*^ and an affine coordinate system *P*^*n*^ as

(4) $$R^{n}\to P^{n}:\begin{array}{cc} \begin{array}{l} {\left(x_{1},x_{2},\cdots ,x_{n}\right)}^{T}\to (x_{1},x_{2},\cdots ,x_{n},1){}^{T}\\ {\left(x_{1},x_{2},\cdots ,x_{n},0\right)}^{T}\to (x_{1},x_{2},\cdots ,x_{n},a){}^{T},a\to 0\\ {\left(x_{1},x_{2},\cdots ,x_{n},a\right)}^{T}\to (x_{1}/a,x_{2}/a,\cdots ,x_{n}/a,1){}^{T},a\to 0 \end{array} & \end{array}$$

where the infinity point (*x*_1_, *x*_2_, ⋯, *x*_*n*_, 0)^T^ is just the limit of (*x*_1_/*a*, *x*_2_/*a*, ⋯, *x*_*n*_/*a*, 1)^T^ under *a* → 0 [18]. Therefore, the infinity point represents the vanishing point of the vision range. It is critical for depth perception of the surrounding scenery that the perspective direction and number of vanishing points are considered when displaying a three-dimensional scene in a two-dimensional plane [19].

When human eyes look into the distance, the fixation point can change in position, and this forms a horizontal vanishing line, as shown in Figure 2. This line is known as the infinity line and is composed of countless vanishing points [20-23]. Similarly, it is also an objective phenomenon that occurs in the visual perception of the external world. It occurs at the intersection of the sky and ground, and provides a broader perspective.

## Results

### Mapping between the scene and visual image

The above brief description of previous research aims to introduce the problem of how a three-dimensional Cartesian coordinate system converted into a two-dimensional plane is able to express a real three-dimensional scene. This also explains why visual images in the retina can provide three-dimensional scene information to an observer. However, how the Cartesian coordinate system in a two-dimensional plane can “faithfully” represent a three-dimensional scene is not known, even though the problem seems trivial. The difference between the stereoscopic perception of actual scenes and a scene in a two-dimensional plane is an important issue in visual information processing, neural computation, psychophysics, and neuroscience, and is also a main research topic in image processing, three-dimensional display methods, and computer vision.

**Figure 3:**
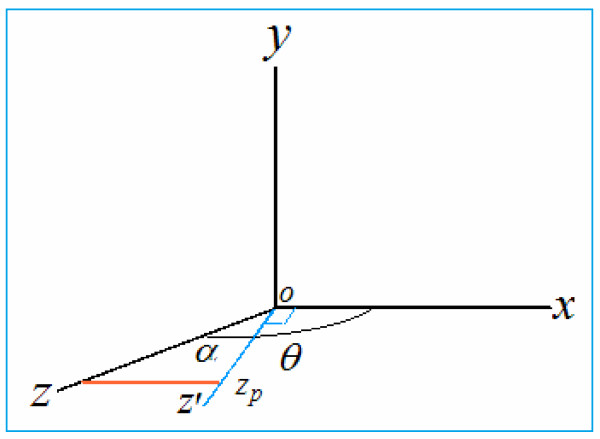
**Cartesian coordinate system on the plane, *α*** **= (*θ*** **- 90**^**∘**^**).**

Figure 3 shows a Cartesian coordinate system. We cannot draw the z axis in the x–y plane such that the included angle between all axes is 90° and the z axis is truly perpendicular to the x–y plane. One approach for drawing the z-axis is to introduce an angle *α* between the external incident light and the optical axis of vision, where *α* ≠ 0. For visual purposes, we can only receive incident light from the front; that is, 0 < *α* is not possible. However, when creating a three-dimensional Cartesian coordinate system to express a stereoscopic image, all quadrants of space should be discussed. Hence, a more general assumption is |*α*| ≠ 0. For the sake of simplicity, we discuss the second quadrant (i.e., 90° < *θ* < 180°). The cases for the other quadrants are the same.

When the angle is within the range 90° < *θ* < 180°, we can express three-dimensional stereoscopic structure in a plane, as shown in Figure 3. According to the statistical results obtained in a psychological experiment that we conducted, when *α* ≈ 30° (*θ* ≈ 120°), the visual stereoscopic perception is strong and the image structure is stable. It can now be *envisaged* that to have a true *z*′ axis perpendicular to the x–y plane (the blue line in Figure 3), the projection of the z axis onto the *z*′ axis is *z* cos(*θ* - 90°) = *z* cos *α*; that is, the projection of the z axis onto the *z*′ axis is simply *z*_*p*_, which is equivalent to the value along the z axis in real three-dimensional space. For example, if *α* = 30°, then

(5) $$z_{p}=z\cos\alpha =z\cos30^{\circ }=\left(\sqrt{3}/2\right)z=0.866z$$

The actual loss of depth information along the z-axis, or the information loss of visual depth perception, is *z*_loss_ = *z* - *z*_*p*_ = *z* - 0.866*z* = *z*(1 - 0.866) = 0.134*z*. Naturally, *α* can have different values and indicate different depths. This is consistent with our experience of visual perception, although we usually pay no attention to it.

As already pointed out, there is a conjugate relation (or causality) between the object point and its image point. When an observer sees a three-dimensional scene I_wr_ = *P*_w_(*x*, *y*, *z*; *V*_*x*_*V*_*y*_*V*_*z*_; *λ*, *t*) in the external world, a corresponding visual image ${\bar{I}}_{\mathrm{rw}}$ = $P_{v}\left(x,y,z^{\prime };V_{x}V_{y}V_{z^{\prime }};\lambda ,t\right)$ forms on the retina that is more than two-dimensional but less than three- dimensional. In turn, if there is a visual image ${\bar{I}}_{\mathrm{rw}}$ on the retina, then the observer perceives the scene of the external world I_wr_ according to ${\bar{I}}_{\mathrm{rw}}$. Hence, the scene and image have the mutually conjugate mapping

$$I_{\mathrm{wr}}\overset{P_{w}\left(x,y,z;V_{x},V_{y},V_{z};\lambda ,t\right)}{\underset{P_{v}\left(x,y,z^{\prime };V_{x},V_{y},V_{z^{\prime }};\lambda ,t\right)}{⇄}}{\bar{I}}_{\mathrm{rw}}$$

(6) $$I_{\mathrm{wr}}\overset{z}{\underset{z^{\prime }}{⇄}}{\bar{I}}_{\mathrm{rw}}$$

$$z^{\prime }↔z\cos\left(\theta -90^{\circ }\right)=z\cos\alpha$$

That is, the actual scene *P*_w_(*x*, *y*, *z*; *V*_*x*_*V*_*y*_*V*_*z*_; *λ*, *t*) is transformed by *z* cos *α* and forms the visual image $P_{v}\left(x,y,z^{\prime };V_{x}V_{y}V_{z^{\prime }};\lambda ,t\right)$ on the retina. It is important to note that, with this transformation relationship, human vision using a two-dimensional image on the retina can perceive an actual three-dimensional scene. According to the above discussion, it is clear that the scene $P_{v}\left(x,y,z^{\prime };V_{x}V_{y}V_{z^{\prime }};\lambda ,t\right)$ can be drawn on a two-dimensional plane, and it can be expresses as a stereoscopic image on the retina and provide stereoscopic perception. The basic concept of this information processing is more clearly expressed as

(7) $$\begin{array}{l} P_{w}\left(x,y,z;V_{x}V_{y}V_{z};\lambda ,t\right)↔P_{v}(x,y,z^{\prime };V_{x}V_{y}V_{z^{\prime }};\lambda ,t)\\ =P_{v}\left(x,y,z\cos\alpha ;V_{x}V_{y}V_{z\cos\alpha };\lambda ,t\right)\\ =P_{v}\left(x,y,z_{p};V_{x}V_{y}V_{z_{p}};\lambda ,t\right) \end{array}$$

That is, through the plenoptic function *P*_w_(*x*, *y*, *z*; *V*_*x*_, *V*_*y*_, *V*_*z*_; *λ*, *t*), I_wr_ forms a visual image ${\bar{I}}_{\mathrm{rw}}$ on the retina. Conversely, the visual image ${\bar{I}}_{\mathrm{rw}}$ matches the external world through the plenoptic function *P*_v_(*x*, *y*, *z*′; *V*_*x*_, *V*_*y*_, *V*_*z*_; *λ*, *t*), and the loss of image information between I_wr_ and ${\bar{I}}_{\mathrm{rw}}$ is approximately *z* cos *α*.

Of course, this is largely a proof of principle, but this discussion demonstrates that it can be used for studies in visual information processing.

It has been confirmed in many eye tracker tests, including psychophysical experiments that the visual system can adjust with eye movements to find a suitable viewing angle and orientation so that the loss of information is minimal [24-26]. This is a fundamental property of the visual system and means that forming visual images on the retina and in the V1 cortex does not require inversion and reconstruction, possibly because the computational cost is too high to solve its inverse, an ill-posed problem without a unique solution.

### Loss of information due to the introduction of a three-dimensional Cartesian coordinate system in the plane

**Figure 4:**
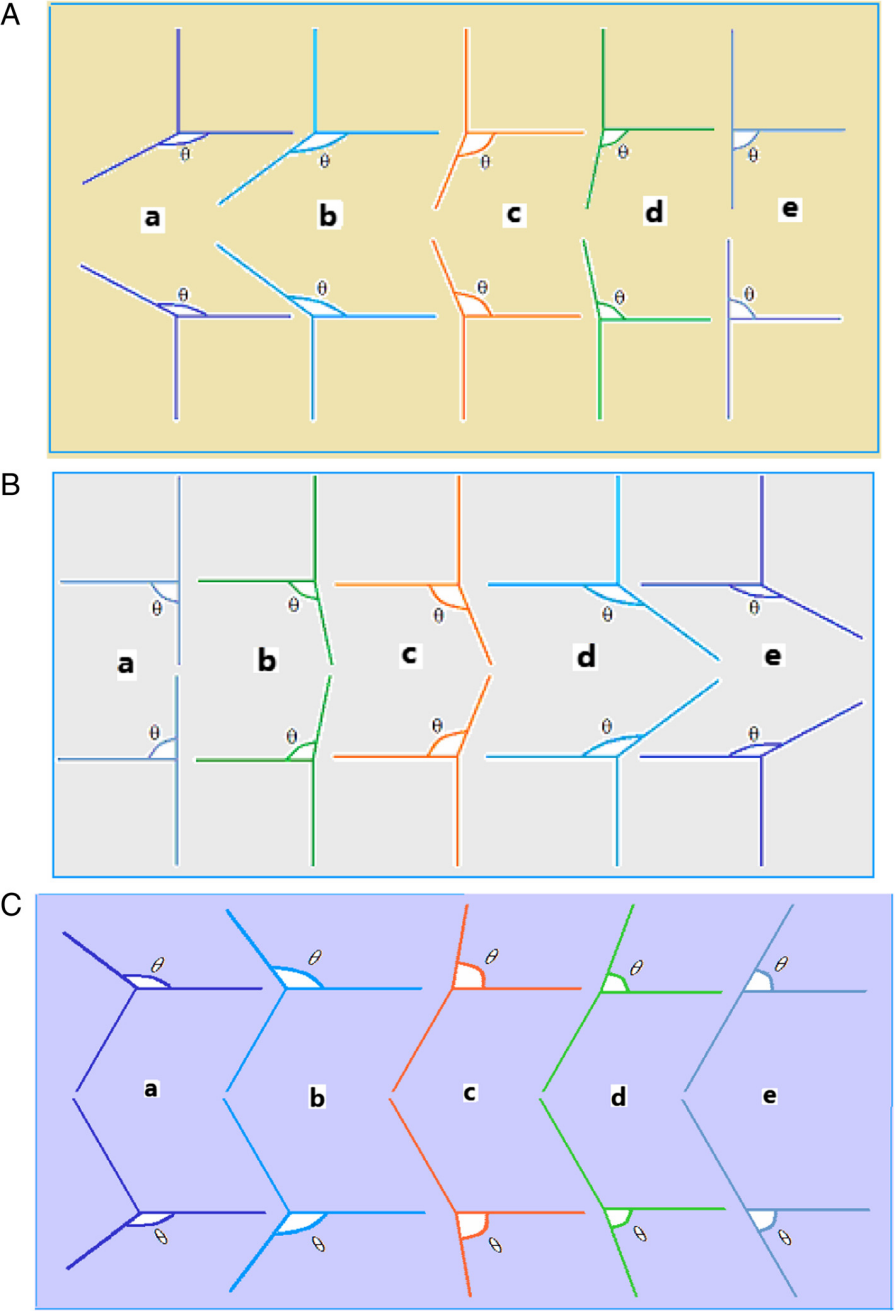
**When the angle *θ *between the x-axis and z-axis is not the same as in the Cartesian coordinate system, the spatial relationships among these axes and visual perception are also different. (A)** The included angle $\cos\alpha =\cos\left(\theta ‒90^{\circ }\right)=\cos\left(30^{\circ }\right)=\sqrt{3}/2=0.866$, angle *θ* from 120° **(a)** to 90° **(e)**. In case **(e)**, there is no stereoscopic perception. **(B)** The included angle *θ* from 90° **a** to 120° **e**. In case **a**, there is no stereoscopic perception. **(C)** The included angle *θ* from 90° **e** to 120° **a**, obtained by rotating **(A)** 90° in the vertical direction and turned 30° in the horizontal direction. In case **(e)**, there is no three-dimensional perception.

Figure 4 shows three groups of three-dimensional Cartesian coordinate systems introduced in a two-dimensional plane. The main differences are the different angles between the x- and z-coordinates; i.e., orientations of the z-axis relative to the x-y plane are different and thus, the stereoscopic visual perception is also different. In the example of Figure 4A (a), when angle *θ* is 120°, the perpendicular relationship among the three axes x, y, and z is most obvious. In Figure 4A (e), when angle *θ* is 90°, there is still a perpendicular relationship among all three axes x, y, and z in the actual space. However, it is not possible to draw a real vertical line perpendicular to the x-y plane itself. It is instead projected points of this vertical line at the coordinate origin by a perpendicular projection into the x-y plane. On the contrary, it can be seen in Figure 4A (a), when angle *θ* is 120°, that the z-axis and the x-y plane and the z-axis and x-coordinate axis have an included angle of 120°. Therefore, it can be considered that, in actual space, its projection in the z axis is $\cos\alpha =\cos\left(\theta ‒90^{\circ }\right)=\cos\left(30^{\circ }\right)=\sqrt{3}/2=0.866$. In other words, the information loss of depth perception is approximately 0.134. In contrast, the Cartesian coordinates can be introduced in the plane and can provide visual stereoscopic perception.

For Figures 4(B) and 4(C), the situation is similar.

### Role of the vanishing point in stereoscopic visual perception

**Figure 5:**
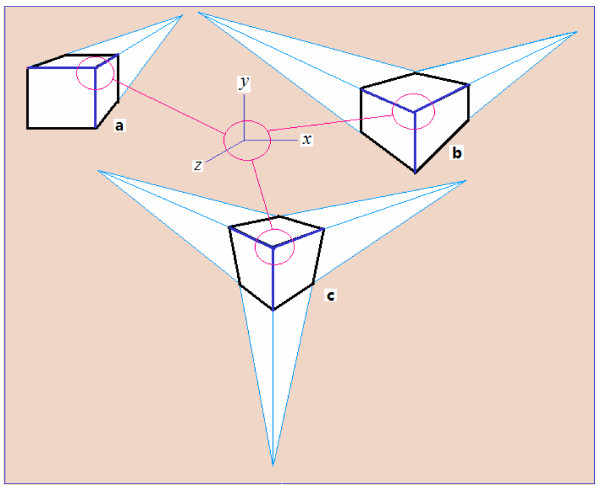
**Vanishing points: (a) one vanishing point, (b) two vanishing points, and (c) three vanishing points.** Each blue cube front marks the Cartesian dimensional rectangular coordinate system of x, y, and z axes and the visual perception of the mutually perpendicular structure between them. In various modern city buildings and green landscapes, photographs and actual scenes from different perspectives can have three vanishing point types.

Figure 5 further illustrates the important role of the vanishing point when introducing a three-dimensional Cartesian coordinate system into a two-dimensional plane to represent a stereoscopic scene. Figure 5(a) is the convergence of projection in a single direction with only a single vanishing point. Figures 5(b) and 5(c) show the convergence of two and three projective directions with two and three vanishing points, respectively. Please note that the blue lines are the x, y, and z coordinates of the Cartesian three-dimensional rectangular coordinate system that can be found in Figures 4(A), 4(B), and 4(C).

The existence of the vanishing point is the fundamental reason why a Cartesian three-dimensional rectangular coordinate system can be drawn in a two-dimensional plane. As mentioned above, it can be easily seen that the formation of vanishing points underlies the optical system of human vision (in principle, see Figure 2). It is also the basis of an affine transformation by which the human visual system is able to perceive the three-dimensional external world, as illustrated in the case of railroad tracks that converge to a single point, forming of a vanishing point (again, in principle, see Figure 2).

### Dual-plane parameterization of the plenoptic function for neural computation of early vision

We know that each pixel of a two-dimensional digital image is a record of the intensity of all light that reaches this point, but does not distinguish between the directions of the light rays. It is just a projection of the light field of the three-dimensional structure, with lost information about phase and direction. Unlike this, the light field refers to the collection of light from any point in space in an arbitrary direction. It comprises all light from different angles that makes a contribution to each pixel. If it takes into account how the angle of light changes with time (*t*), it is a dynamic light field. The plenoptic function is a good mathematical description of the dynamic light field. However, questions remain regarding how the human visual system perceives and processes the structural information of the dynamic light field as well as how it receives three-dimensional information from the image on the retina.

Studies by Zeki, Livingstone et al. have indicated that in the human visual system color information is transmitted in a separate channel in the cerebral cortex [27-29]. Therefore, wavelength λ can be separated from the plenoptic function. In addition, position, direction, and orientation information can also be separated. In this way, without considering time variation and separating dimensions, the seven-dimensional plenoptic function *P*_v_ = *P*_v_(*θ*, *ϕ*, *λ*, *t*, *V*_*x*_, *V*_*y*_, *V*_*z*_) can describe and reconstruct plenoptic images, or visual information of the objective world with different combinations of variables.

**Figure 6:**
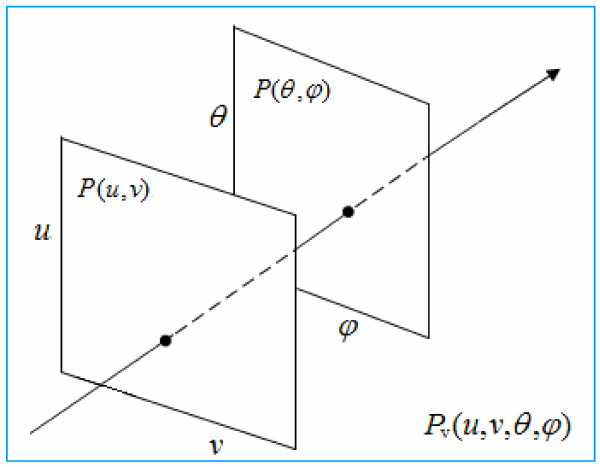
**Nested representation of the dual-plane *P*(*θ*,** ***φ*) and *P*(*u*,** ***v*) parameterization for the retina and the primary visual cortex.**

When the viewer’s eyes are looking at a point in any scene, emitted or reflected light rays from this point will enter the eye. The intensity information of the incident light ray carried in Vx, Vy, Vz is received by the eye. Since the optical and the coordinate Z axes are the same, the light intensity of the stimulus is converted into the strength of photosensitive cell activity. Therefore, only angles *θ and*φ of the light need to be recorded. For this reason, the plenoptic function can be parameterized using a dual-plane representation formed from *P*(*u*, *v*) and *P*(*θ*, *φ*), as shown in Figure 5. A light ray intersects with the position plane *P*(*u*, *v*) and angle plane *P*(*θ*, *φ*) at (*u*, *v*) and (*θ*, *φ*), respectively. The coordinates of the points of intersection (*u*, *v*) and (*θ*, *φ*) can be used to describe this plenoptic function. The form of a two-plane parameterization is very simple and intuitive. Hence researchers have used this method for visualization of light field data, namely, using double nested coordinates to arrange the data of a four-dimensional light field into a two-dimensional plane, forming two symmetrical representations. Figure 6 is an example in which *P*(*θ*, *φ*) is the inner angle plane and *P*(*u*, *v*) is the outer position plane. As can be seen, a light ray at different angles corresponds to different viewing angles in the imaging plane. Therefore, such representation of the light field may be closely related to a neural representation of the retina and primary visual cortex of the human visual system. Many experiments in neurobiology have shown there are topological mappings with a one-to-one correspondence between the retina and the V1 cortex that is established through projections from the ganglion cells via the lateral geniculate nucleus to the primary visual cortex. Through photosensitive cells, the retina records the position information of the incident light ray, while the V1 cortex processes the orientation information through simple and complex cells, as well as the orientation function columns [30-32]. Therefore, for early visual information processing, this is a viable solution. It minimizes the loss of information as much as possible without making the algorithm too complex. Of course, to do this is not an easy matter, and whether the human visual system employs this strategy needs further study.

### Three-dimensional visual perceptions of images in a two-dimensional plane

We know that if an image of a scene on a plane does not contain depth information, the human visual system has no way of perceiving the scene three-dimensionally. When observing the external world, human vision has characteristics of perceptual constancy (e.g., size, color, and shape constancy). This constancy is the basis of an affine transformation, which depends on vanishing points and vanishing lines in visual perception and is determined by the characteristics of the optical system of the visual pathway. As Rock pointed out, the height of an object in the base plane is an important depth cue. It can be calculated according to [33-35]

(8) S=δAD

where *S* is the height of the object on the fundamental plane (i.e., on the x–z plane in Figure 2), *δ* is the viewing angle of the camera, *D* is the distance (i.e., distance along the z-axis) between the photographer and the object, or the depth information, and *A* is the scaling factor of the retina. Formula (8) is used to reconstruct a three-dimensional scene from an image in a two-dimensional plane. Figure 2 is an optical model of the affine transformation of the retina.

**Figure 7:**
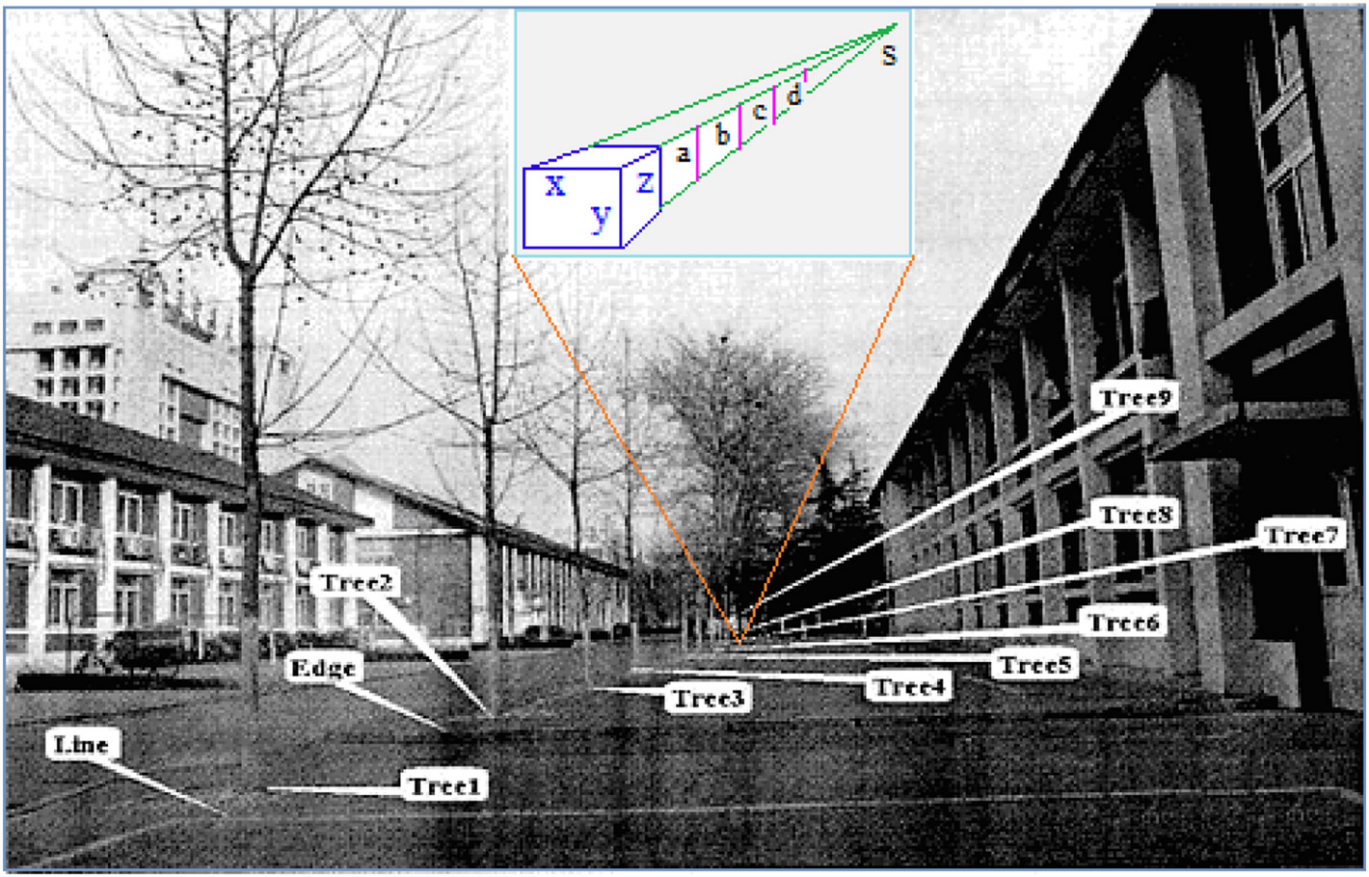
**The image plane was tilted and the camera height was 0.87 m.** The location of the picture is the front of the No. 8 student dormitory building at Beijing Jiaotong University [37]. The inset in Figure 7 shows size constancy of visual perception, in this inset there is only one vanishing point “s”. Obviously, the size of **a**, **b**, **c**, **d** are almost proportionately reduced, which reflects linear property of size constancy, and from this linear property we can calculate depth distances of these trees in the Figure 7.

For the sake of simplicity, we analyze only the example (taken from the literature [36,37]) of one vanishing point, as shown in Figures 7(a) and 7(b).

The main purpose of the calculation example is to show that we can use the vanishing point, size constancy and affine transformation model in Figure 2 to calculate the depth value in a picture taken of an actual scene. A comparison of the calculation results with actual measurements reveals that the vanishing point reflecting the basic characteristics of the optical system of human vision and size constancy reflecting cognitive psychological characteristics are important in accessing depth information in a two-dimensional picture.

**Figure 8:**
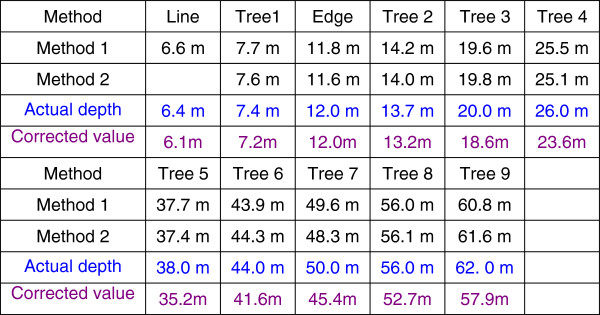
**Computation results of depth distance of trees in Figure**7**according to the size constant of visual perception.**

The example focuses on the absolute depth perception of white markers, edges on the ground and nine trees (see Figure 7). Comparisons with measurements are listed in Figure 8.

Specific calculations are carried out employing two methods. The first method employs psychological methods based on formulae (8) and (9), and the second method employs an affine transformation based on an optical model of vision (Figure 2). Known parameters required for the calculation are the height of the camera from the ground (0.87 m) and the horizontal distance between the photographer and first white line on the ground (see Figure 7) (D = 6.40 m). The camera is a Nikon-E3700CCD, and the image size is 2048 × 1536 pixels. The calculation includes the vanishing point, the vanishing line, the height of the tree, and the line whose change in depth value is fastest on the ground portion of the image plane. Specific calculations are found in the literature [36,37]. Naturally, algorithms of computer vision can also be used [38-42].

The results of both calculation methods are consistent with actual measurement results, showing that the calculation methods are reasonable and reflect the consistency between visual psychology and the optical system of visual pathways in the depth perception of an actual scene. More importantly, the results show that a two-dimensional image can contain rich three-dimensional information that is perceived by the visual system itself.

We know that when looking at an image or a scene from different angles, the perceived depth of field changes. To show depth information provided by constancy and the affine transformation in a two-dimensional image plane (see the model in Figure 2), formula (8) is corrected according to equation (6), such that the image height of the object may be calculated according to

(9) $$S=\delta \cos\mathrm{\alpha AD}=\delta \cos\left(\theta -90^{\circ }\right)\mathrm{AD},$$

where *α* = (*θ* - 90^∘^) is the included angle between the z-axis and *z*′ -axis (namely the optical axis or gaze direction, see Figure 3) when looking at the image. Hence, we use formula (9) to correct the result of the depth information given by method 2, and these corrected values are also given in Figure 8. After taking into consideration information loss, the corrected value roughly reflects the visual depth perception obtained from the image (or two-dimensional plane).

The proposed method is completely different from three-dimensional image reconstruction that uses binocular disparity and corresponding points in the field of visual computational theory, or three-dimensional reconstruction using corresponding points in two images taken by two cameras in the field of computer vision. The processing method of visual perception has advantages [36,43] such as efficiency, robustness, and low computational complexity. It is therefore worthy of study by researchers in the fields of computer vision and visual neural computational theory.

In Appendix 1, according to Figure 2, Figure 3, the formulae (7), (8) and (9) we will make some predictions about stereoscopic perception of the image on a two-dimensional plane, including: 1 The picture, in which there is no vanishing point; 2 Alternating process of Cartesian coordinate system and affine coordinate system; 3 The Moon Illusion, and 4 The inversion reconstruction of visual image.

## Discussion

This article explores how the human vision system extracts depth information from an image of a scene in a Cartesian rectangular coordinate system on a two-dimensional plane. We introduced the concepts of a plenoptic function in the optical system of the visual pathway. In the section of methods “Computational approach in visual cortex V1”, we proposed an algorithm of coincidence test, in which an image primitive *r*_*U*,*V*_(*a*) transferred by ganglion cells from retina to visual cortex V1 will coincide with neurons’ receptive field [*B*_*θ*,*φ*_(*g*)]_*Θ* × *Φ*_ in cortical columns.

Note that, all of neurons in the columns simultaneously carry out compliance testing operations in parallel manner, neuron of [*B*_*θ*,*φ*_(*g*)]_*Θ* × *Φ*_, which most consistent with the image primitives *r*_*U*,*V*_(*a*), is activated and its firing rate is strongest, so that each image primitive *r*_*U*,*V*_(*a*) can be detected. Because it is distributed and parallel processing (see following equation 12), the mathematic operation of coincidence test is very simple, robust, fast and completely consistent with the pattern of stimulating → firing → response of neurons.

Based on the biological function and structure of the visual pathway and the primary visual cortex, we proposed the dual-parameterized method, which can be expressed as *P*(*u*, *v*) ⊗ *P*(*θ*, *φ*), and is mathematically equivalent to the formula *P*_v_(*u*, *v*; *θ*, *φ*) = [*R*_*u*,*v*_(*a*)]_*U* × *V*_ ⊗ [*B*_*θ*,*φ*_(*g*)]_*Θ* × *Φ*_, or to formula 12, as described as follows.

In this paper, we have raised an issue “in the two-dimensional plane, why can three-dimensional structure of a picture be expressed by adopting Cartesian coordinate system?”, its importance is to study the information processing from 2D retinal image to three-dimensional visual perception. Based on neural computation of visual cortex V1, and taking into account the affine transformation processing of visual image information and size constancy of visual perception, and also considered the findings of psychophysics. However, formula (8) and Figure 2 show that the psychology of visual perception can explain how the human vision perceives a three-dimensional scene from a two-dimensional retina. Because of a structured light field that densely fills the surroundings, human vision processes information according to formulae (6) and (7). The information loss from the three-dimensional scene in the external world to a visual image in the two-dimensional retina is small, and hence the visual image on the retina contains the rich information of the three-dimensional scene. Therefore, we may consider the visual system as a causal system, meaning that the scene has a one-to-one correspondence with the visual image. The scene produces a visual image in the retina, and conversely, if a visual image is formed in the retina, then a viewer perceives the external scene that produced that visual image in the retina.

We know the reconstruction of visual image is just a hard inverse problem as a major topic of research in computer vision, its concern is how to use binocular disparity information (i.e., corresponding point in dual camera image) to find a stable and efficient reconstruction algorithms; it is also an issues concerned by current 3D display technical, its focal point is that this kind of research will able to provide an effective method for better 3D display technology; of course, it is also hard problem to trouble the research of biological vision, vision research mainly is to start from unified basic viewpoint of the biological function and structure of the vision and then explore how to achieve the following information processing by human visual system, namely : from retinal images of three-dimensional scenes to → 2D visual image, and to → 3D visual perception. In the first section “Mapping between the scene and visual image ” of this paper, this issue has been discussed in more detail, in which the formulas (6) and (7) had shown that there is no specific reconstruction algorithm from 2D retinal images to three-dimensional scene. At present, to an image, the processing time of the brain has been determined by using an approach of rapid serial visual presentation of image series and cognitive psychological method, it is just 13 ms [44]. So fast processing speed shows that human vision may not be obtained three-dimensional depth perception by using reconstruction method based on the corresponding point, because this method and related algorithms are too complicated, the computational cost is also too high, for this reason, it is impossible to implement such a reconstruction algorithms by using the neurons, neural circuits and partial network. This paper studies how to obtain stereoscopic visual perception, when viewing pictures on the plane, obviously, this issue has important significance for vision information processing; of course, it is also the same for computer vision.

According to Figures 2 and 3, the formulae (7), (8) and (9) we may make some predictions about stereoscopic perception of the image on a two-dimensional plane, including:

1. The picture, in which there is no vanishing point;

2. Alternating process of Cartesian coordinate system and affine coordinate system; 3. The Moon Illusion (see Appendix 1 for details [45]).

We have reason to believe that rough outline of theory about three-dimensional visual perception of visual pathway is generally clear.

## Conclusion

We know that there are many monocular depth cues (e.g., perspective scaling, linear perspective, texture gradient, atmospheric perspective, occlusion, light and shade, color, and image hierarchy structure) that can also form depth perception. However, in this paper, we study how to express stereoscopic visual perception in a two-dimensional plane and only use the parameterized method of a dual plane of the plenoptic function to process the visual information of an image.

According to the principle of graceful degradation proposed by Marr [5], if the visual system calculates a rough two-dimensional description from an image, it will be able to calculate a rough three-dimensional description represented by this image. In other words, human vision can perceive the real three-dimensional description from stereoscopic images on a two-dimensional plane. Marr posed the problem in this way: “The contours of the image are two-dimensional, but we often come to understand these contours from the perspective of three dimensions. Therefore, the key question is how do we make a three-dimensional interpretation of the two-dimensional contour? Why can we make this explanation?”

We have studied this issue, and to answer Marr’s question, this paper presents a preliminary explanation. The main results are as follows:

1. Two different plenoptic functions to describe the objective world were introduced. The difference between these two functions *P*_w_ and *P*_v_ regarding the external scene obtained by visual perception were analyzed, and their specific applications in visual perception were discussed.

2. The main results were how the processing of visual depth information perceived in stereoscopic scenes can be displayed in a two-dimensional plane. Constraints for the coordinates and an algorithm implementation were also provided, in particular, a method used to separate the plenoptic function and a transformation from the retina to the visual cortex. A dual-plane parameterized method and its features in neural computing from the visual pathway to visual cortex V1 were discussed. Numerical experiments showed that the advantages of this method are efficiency, simplicity, and robustness.

3. Size constancy, a vanishing point, and vanishing line form the psychophysiological basis for visual perception of the external world, as well as the introduction of the three-dimensional Cartesian rectangular coordinate system into a two-dimensional plane. This study revealed the corresponding relationship between perceptual constancy, the optical system of vision, and the mapping of the vanishing point and line in the visual image on the retina.

The main results of this paper are a preliminary explanation as to why and how the Cartesian rectangular coordinate system can be introduced into a two- dimensional plane, and how a three-dimensional scene can be perceived in a two-dimensional plane. The results of this study are of significance in visual depth perception and possibly in applications of computational vision.

## Methods

### Computational approach in visual cortex V1

**Figure 9:**
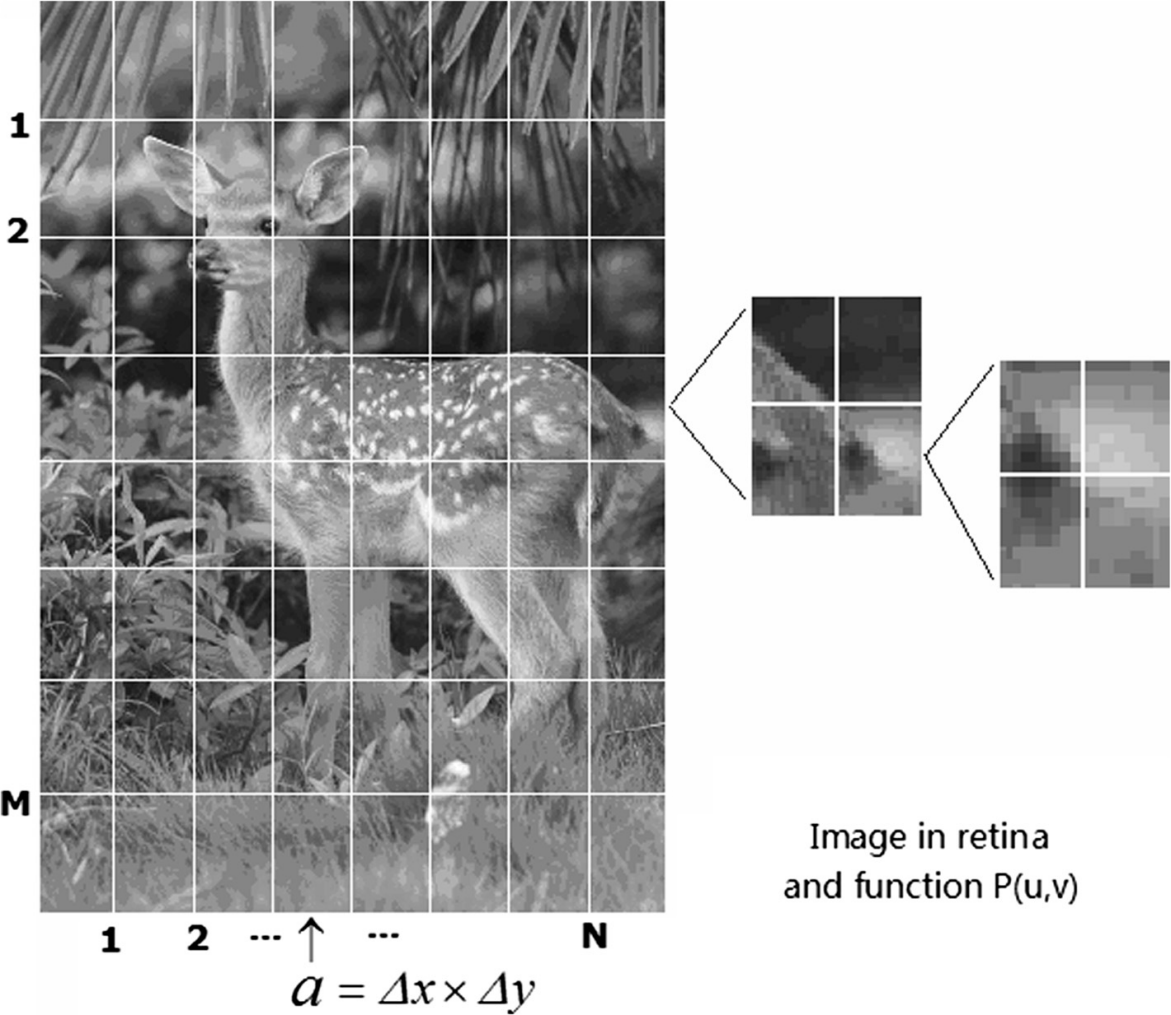
**The retina –p(u, v) plane is divided into M × N = 10**^**6 **^**patches according to the receptive field sizes of ganglion cells.** A simpler approach is based on the complexity of the image, that is, according to the distribution of basic characteristics (line, corners, and curves for example) in an image. An A4-sized image can be divided into 128 or 64 patches in the first stage, and at the second stage, for pitch of the image can be divided by 8, 16, and so on. It is worth noting that when the total number of first-stage divisions is large, the total number of second-stage divisions should be small [46].

The adopted dual plane parameterized representation makes the mathematical form of the visual pathway and primary visual cortex neural computation more concise and intuitive. More specifically, the retina may be represented by the plane *P*(*u*, *v*). A light intensity array of external stimuli is able to form an image on the retina when observing the surrounding world. Usually, a visual image is independently transferred to the lateral geniculate nucleus (LGN) through ganglion cells, reaches cortex V1, and is reproduced in V1. Obviously, every image patch is transferred through one channel. Suppose the number of channels is M × N, meaning that the visual image is divided into M × N patches. When dividing an image, for convenience, a rectangular rather than circular receptive field of ganglion cells is assumed, and it is also generally assumed that the size of the receptive field of a ganglion cell is *a* = *Δx* × *Δy*, whose area is approximately equal to 10 × 10 μm. It is known that the total number of ganglion cells is 10^6^, hence, M × N ≈ 10^6^. As pointed out in [46], each patch is assumed to have the same size (α) as the receptive field of a ganglion cell, namely, the visual image is divided into M × N units. If the area of the whole image is A, and every channel only has one patch of A, then, A = (M × N) α = (M × N) *Δx* × *Δy* = 10^6^*Δx* × *Δy*. This is the easiest way to divide an image. This division becomes different for an image that has a different scale. In practice, the sizes of *Δx* and *Δy* are mainly dependent on the resolution of the image (or pixel density). A more convenient approach is based on the minimum size of the pixels in the display device for dividing images of different sizes. The current minimum pixel size is approximately 0.2 mm (200 microns). Therefore, as the image scale is increased, the size of *Δx* and *Δy* also increases. Then, according to the size of the receptive field of ganglion cells, the visual image on the retina, that is, the plane *P*(*u*, *v*), is divided into M × N patches (or image primitives), as shown in Figure 9[47,48].

The entire image in the retina can be represented using the following matrix:

(10) $$\begin{array}{cc} {\left[R_{u,v}\left(a\right)\right]}_{U\times V}=\left[\begin{array}{cccc} r_{1,1}\left(a\right) & r_{1,2}\left(a\right) & \cdots & r_{1,V}\left(a\right)\\ r_{2,1}\left(a\right) & r_{2,2}\left(a\right) & \cdots & r_{2,V}\left(a\right)\\ \vdots & \vdots & \vdots & \vdots \\ r_{U,1}\left(a\right) & r_{U,2}\left(a\right) & \cdots & r_{U,V}\left(a\right) \end{array}\right],\\ u=1,2,\cdots ,U,\cdot \cdot \cdot v=1,2,\cdots ,V & \end{array}$$

Ganglion cells transmit a neural firing spike train to the LGN. Then, similarly, magnocellulars and parvocells in the LGN transmit information about the image patches into 4C_α_ (magnocellular layer) and 4C_β_ (parvocellular layer) of the fourth layer in the V1 cortex. Naturally, these coded neural firing spike trains need to be decoded and information about their image primitives need to be restored. A neural decoding circuit with 40 Hz synchronous oscillation accomplishes this task [49].

In cortex V1, the shapes of a receptive field of the simple and complex cells are bar-shaped patterns of orientation and bandwidth selectivity. The sizes of the receptive field of the simple and complex cells are about 20–50 μm. Their orientation and maximum resolutions are about 10° and 0.25°, respectively. Hence, their line resolution is between 5.0–100 μm [9].

**Figure 10:**
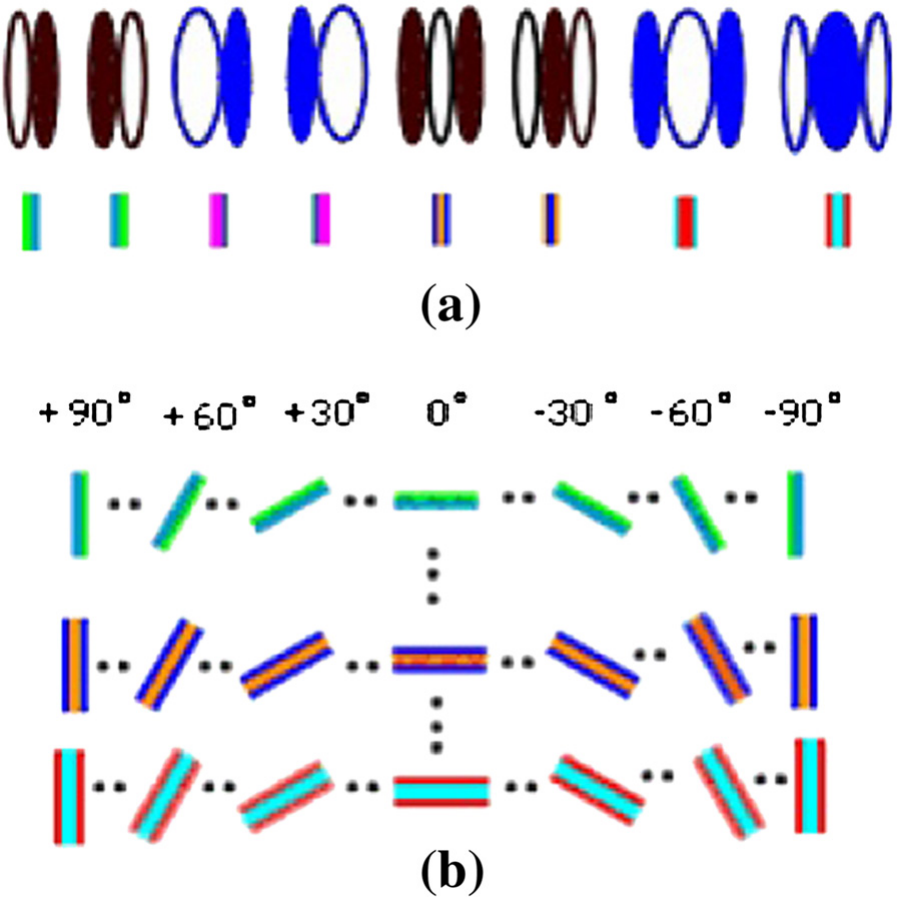
**V1 cortical columns as the basic components of the information processing unit. (a)**. Neuron receptive fields with eight kinds of typical shapes. **(b)**. the functional column from 0° to 180° divided into 18 different orientations at 10° intervals [9,46].

Accordingly, the V1 cortex is represented by the plane *P*(*θ*, *φ*). From neurophysiology and neuroanatomy [30-32], it is known that the V1 cortex is organized in functional modules orthogonal to the cortical layers. Each module contains two functional columns, one is the left eye dominant column and the other is the right eye dominant column. It is reasonable to assume that these functional columns have the same information processing functions. Each functional column consists of many receptive fields with different orientations and frequencies [32,48]. Therefore, receptive fields in the functional column can be expressed in a matrix form. Hence, 18 function columns represent orientations from 0° to 180°, 10° apart, and are arranged in a row. Every orientation consists of a total of eight kinds of typical receptive fields (composed of bio-orthogonal Gabor wavelets with different frequencies) arranged in eight rows, as shown in Figure 10[46]. Of course, for a more detailed description, additional receptive fields can be added.

Similarly, the functional column shown in Figure 9 may be expressed in a matrix form as:

$${\left[B_{\theta ,\varphi }\left(g\right)\right]}_{\Theta \times \Phi }=\left[\begin{array}{cccc} b_{1,1}\left(g\right) & b_{1,2}\left(g\right) & \cdots & b_{1,\Phi }\left(g\right)\\ b_{2,1}\left(g\right) & b_{2,2}\left(g\right) & \cdots & b_{2,\Phi }\left(g\right)\\ \vdots & \vdots & \vdots & \vdots \\ b_{\Theta ,1}\left(g\right) & b_{\Theta ,2}\left(g\right) & \cdots & b_{\Theta ,\Phi }\left(g\right) \end{array}\right]$$

(11) θ=1,2,⋯,Θ,⋅⋅⋅φ=1,2,⋯,Φ

where (*g*) is obtained from a numerical simulation or calculation using a Gabor wavelet function. The main function of the neural decoding circuit is to extract information from each image patch from the neural firing spike trains, after encoding and decoding to restore the original visual image. For brevity, these intermediate steps are not considered, so that the entire information processing process can be represented as a Kronecker product ⊗ between matrixes [*R*_*u*,*v*_(*a*)]_*U* × *V*_ and [*B*_*θ*,*φ*_(*g*)]_*Θ* × *Φ*_, given by

$$P_{v}\left(u,v;\theta ,\varphi \right)={\left[R_{u,v}\left(a\right)\right]}_{U\times V}⊗{\left[B_{\theta ,\varphi }\left(g\right)\right]}_{\Theta \times \Phi }=\left[\begin{array}{cccc} r_{1,1}\left(a\right) & r_{1,2}\left(a\right) & \cdots & r_{1,V}\left(a\right)\\ r_{2,1}\left(a\right) & r_{2,2}\left(a\right) & \cdots & r_{2,V}\left(a\right)\\ \vdots & \vdots & \vdots & \vdots \\ r_{U,1}\left(a\right) & r_{U,2}\left(a\right) & \cdots & r_{U,V}\left(a\right) \end{array}\right]⊗{\left[B_{\theta ,\varphi }\left(g\right)\right]}_{\Theta \times \Phi }\left|{}_{\text{max}}\right)=$$

$$\left[\begin{array}{cccc} r_{1,1}\left(a\right){\left[B_{\theta ,\varphi }\left(g\right)\right]}_{\Theta \times \Phi } & r_{1,2}\left(a\right){\left[B_{\theta ,\varphi }\left(g\right)\right]}_{\Theta \times \Phi } & \cdots & r_{1,V}\left(a\right){\left[B_{\theta ,\varphi }\left(g\right)\right]}_{\Theta \times \Phi }\\ r_{2,1}\left(a\right){\left[B_{=\theta ,\varphi }\left(g\right)\right]}_{\Theta \times \Phi } & r_{2,2}\left(a\right){\left[B_{\theta ,\varphi }\left(g\right)\right]}_{\Theta \times \Phi } & \cdots & r_{2,V}\left(a\right){\left[B_{\theta ,\varphi }\left(g\right)\right]}_{\Theta \times \Phi }\\ \vdots & \vdots & \vdots & \vdots \\ r_{U,1}\left(a\right){\left[B_{\theta ,\varphi }\left(g\right)\right]}_{\Theta \times \Phi } & r_{U,2}\left(a\right){\left[B_{\theta ,\varphi }\left(g\right)\right]}_{\Theta \times \Phi } & \cdots & r_{U,V}\left(a\right){\left[B_{\theta ,\varphi }\left(g\right)\right]}_{\Theta \times \Phi } \end{array}\right]\left|{}_{\text{max}}\right)$$

(12) $$=\left[\begin{array}{cccc} r_{1,1}\left(a\right) & r_{1,2}\left(a\right) & \cdots & r_{1,V}\left(a\right)\\ r_{2,1}\left(a\right) & r_{2,2}\left(a\right) & \cdots & r_{2,V}\left(a\right)\\ \vdots & \vdots & \vdots & \vdots \\ r_{U,1}\left(a\right) & r_{U,2}\left(a\right) & \cdots & r_{U,V}\left(a\right) \end{array}\right]⊗\left[\begin{array}{cccc} b_{1,1}\left(g\right) & b_{1,2}\left(g\right) & \cdots & b_{1,\Phi }\left(g\right)\\ b_{2,1}\left(g\right) & b_{2,2}\left(g\right) & \cdots & b_{2,\Phi }\left(g\right)\\ \vdots & \vdots & \vdots & \vdots \\ b_{\Theta ,1}\left(g\right) & b_{\Theta ,2}\left(g\right) & \cdots & b_{\Theta ,\Phi }\left(g\right) \end{array}\right]\left|{}_{\text{max}}\right)$$

The neurobiological significance of the Kronecker product ⊗ between the two matrixes [*R*_*u*,*v*_(*a*)]_*U* × *V*_ and [*B*_*θ*,*φ*_(*g*)]_*Θ* × *Φ*_ lies in the assumption that these functional columns have the same information processing function and each functional column consists of many receptive fields with different directions and frequencies [46,49]. The processing of the visual image in the retina and the corresponding points in the V1 cortex, in essence, is a process in which all receptive fields with different orientations in the cortical columns select suitable image patches. Those that correspond to the most active neurons are selected. This assumption is in accordance with the experimental results of the function and structure of the V1 cortex [50].

According to Figure 10 and formula (10), the total orientation of 180° is divided into 18 intervals, thus the orientation resolution of the human vision is only 10°. In fact, the resolution is much higher than 10° and is actually down to 0.25°. This is because the brain applies an interpolation method between the adjacent optimal orientations. In other words, when the preferred orientation of the receptive field of a cortical simple cell is close to the optimal orientation, a weighted average value based on the number of activated simple cells is calculated [9,46,48,51]. Performing a numerical simulation based on formula (10), the azimuth angle in Figure 10 may be divided more finely, at the same time increasing the type and number of the receptive fields in formula (10). According to the complexity of the visual image, for example, the number of image features (line, corners, and curves for example) and their distribution density, the total number of blocks (primitives) can be determined (first level division), and then the number of sub-blocks is determined (secondary level division). If necessary, the sub-blocks can also be divided. The purpose of doing this is that one can simulate multi-scale properties of the visual system. In addition, it could make the results of numerical simulations more accurate, as the error between the source image (visual image) and results of numerical simulation would be smaller.

## Appendix

### Appendix 1 of the section 5 of text

From Figures 2 and 3 and formulae (7), (8) and (9), we make predictions about the stereoscopic perception of an image on a two-dimensional plane.

### Image without a vanishing point

**Figure 11:**
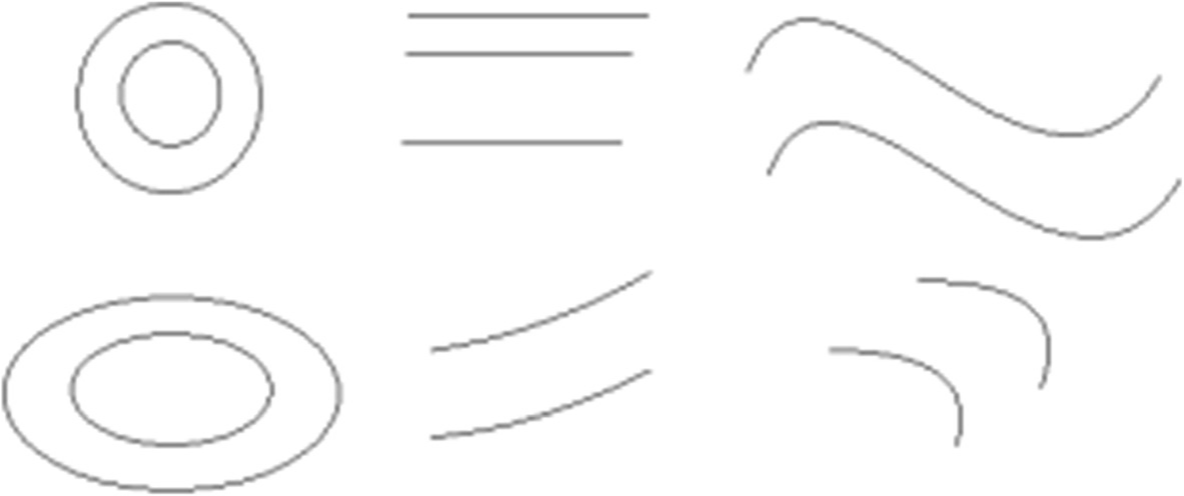
Graphs with no vanishing points, depth cues or stereoscopic information.

In a typical case, there are one, two or three vanishing points in a scene graph, as shown in Figure 5. If there is no vanishing point in a picture, then there is no intersection of line segments on the plane of the graph; there are only a variety of parallel lines or curves with different directions and different shapes. This is illustrated in Figure 11 for a few typical graphs. In fact, there are many different graphs, lines or curves in a picture, but they do not intersect in the case considered. Obviously, Figure 11 does not contain any stereoscopic information; therefore, human vision cannot obtain any three-dimensional perception or depth information. The reason is simply that there is no vanishing point S, and the model of affine transformation (see Figure 2) does not hold well in this case.

Alternating use of a Cartesian coordinate system and affine coordinate system

According to Equation (13),

(13) $$R^{n}\to P^{n}:\begin{array}{cc} \begin{array}{l} {\left(x_{1},x_{2},\cdots ,x_{n}\right)}^{T}\to (x_{1},x_{2},\cdots ,x_{n},1){}^{T}\\ {\left(x_{1},x_{2},\cdots ,x_{n},0\right)}^{T}\to (x_{1},x_{2},\cdots ,x_{n},a){}^{T},a\to 0\\ {\left(x_{1},x_{2},\cdots ,x_{n},a\right)}^{T}\to (x_{1}/a,x_{2}/a,\cdots ,x_{n}/a,1){}^{T},a\to 0 \end{array} & \end{array}$$

**Figure 12:**
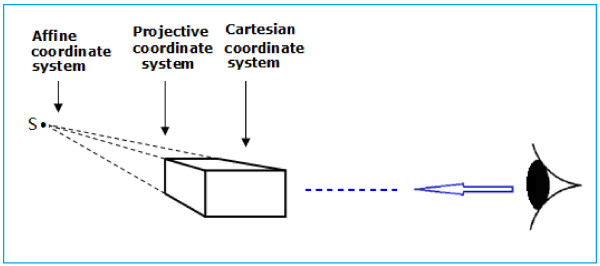
Relationship among the Cartesian coordinate system, projective coordinate system and affine coordinate system.

The mapping from a Cartesian coordinate system to an affine coordinate system is a gradual process in which *α* → 0; i.e., when the distance between an observer and his/her fixation point or spatial range of visual gaze is very small, the Cartesian coordinate system plays a major role. As *α* → 0, or the fixation point goes into the distance, an affine coordinate system instead of a Cartesian coordinate system comes into play, and parallel lines gradually converge to a point that is simply the vanishing point (Figure 12).

**Figure 13:**
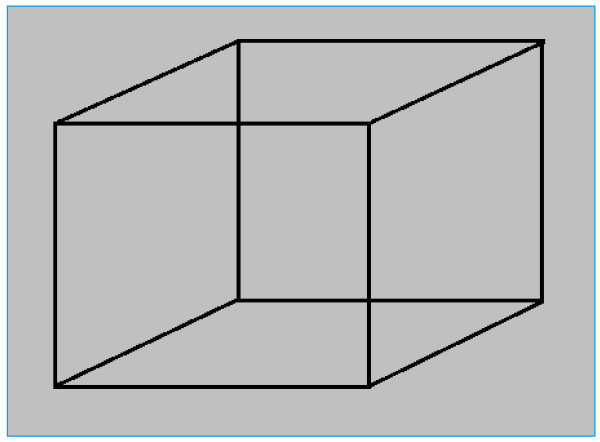
Reversion phenomenon and three-dimensional visual perception of a Necker cube.

The inversing of a Necker cube, which is a known problem of stereoscopic perception, can be explained by the alternating of a Cartesian coordinate system and affine coordinate system. The Necker cube has a constant perspective angle; i.e., each of the four sides of a Necker cube (see Figure 13) in the vertical direction, horizontal direction and tilt direction are parallel to each other.

There seems to be no vanishing point in Figure 13. In fact, each of the four parallel sides extends to infinity in the left and right, up and down, and forward and backward directions. The parallel sides converge together and inevitably form vanishing points, all of which form a closed circle. This closed circle is the vanishing line. The circular vanishing line is the fundamental reason why the human’s visual perception can invert opposite sides for the front and back in Figure 13. In Figure 13, the Necker cube is consistent with the representation in Figure 3. As this representation can generate three-dimensional perception, also in line with the representation in Figures 2 and 5, it is not repeated.

### Moon Illusion

**Figure 14:**
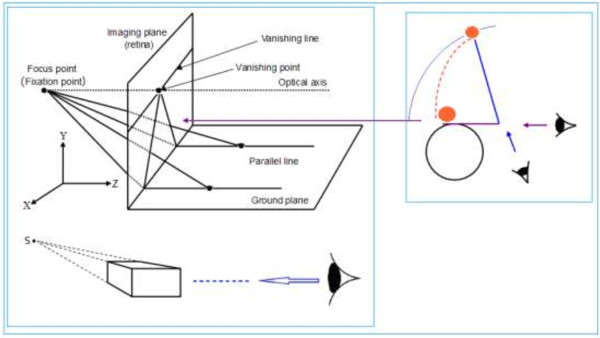
Schematic diagram of the Moon illusion.

The Moon and Sun appear larger on the horizon than at zenith, which is a phenomenon known as the Moon illusion. There are many research findings and interpretations for this problem. However, we believe that the Moon and Sun on the horizon are simply on the lower part of the vanishing line in Figure 2; i.e., the ground portion of Figure 2. Because the horizon is in the distance, the angle of the viewer’s gaze is very small, and the horizon is much lower, close to the bottom of the ground portion in Figure 2. The resulting depth perception is much smaller than that if the vanishing line is in the central portion of Figure 2. When the observer is looking at the sky, his/her visual field of view is about 150° in the vertical direction, and therefore, the observer sees the Moon (or Sun) and, at the same time, the distant horizon and near ground (Figure 14) as a reference point with which to estimate the distance between the Moon (or Sun) and the observer. Obviously, this distance is much greater than the distance when watching the Moon on the horizon.

Because of the combined effects of visual perception’s constancy and vision’s optical property of far objects being smaller and near objects being larger, the Moon (or Sun) in the sky is perceived to be further from the observer, and area of the Moon is thus perceived to be smaller. Existing experimental and calculation results are that the Moon on the horizon is visually perceived to be 1.5 to 1.7 times as large as that in the sky [1,2].

## Competing interests

The authors declare that they have no competing interests.

## Authors’ contributions

ZS proposed and conceived the study and wrote a first draft. QJ, ZQ, and LC took part in designing the study and contributed to the comparative analysis. LX and SS took part in the numerical calculations, verification and analysis of the data and drew all the illustrations. All authors discussed and modified the revised manuscript and all authors have accepted the final version.

## Supplementary Material

Additional file 1 **Straight iron rod passes through two mutually perpendicular nuts in a way impossible in a real scene (**http://yyyggg1398.blog.163.com/blog/static/102113077201041610523293/**).** Click here for file

Additional file 2 Visual depth perception in an image of a truss structure. Click here for file

Additional file 3 Visual depth perception in a landscape image. Click here for file

Additional file 4 Three-dimensional scene with stereoscopic visual perception indicating a range of depth at the Metropolitan Museum of Art, New York. Click here for file

Additional file 5 **Vivid effect of three-dimensional perception in a picture painted on the pavement [**[9]**] (**http://yyyggg1398.blog.163.com/blog/static/1021130772010416104031212**).** Click here for file
