# Supplementary figures and images for: The representation of visual depth perception based on the plenoptic function in the retina and its neural computation in visual cortex V1
Source: BMC Neurosci. 2014 Apr 23;15:50. doi: 10.1186/1471-2202-15-50 (PMC4023552; doi:10.1186/1471-2202-15-50)

Attached picture 1

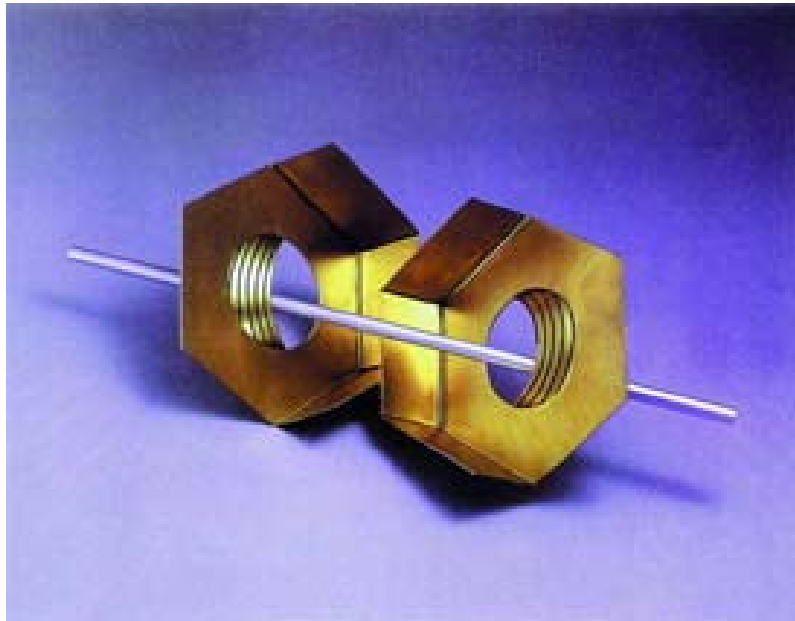

Supplement: Additional file 1 — Straight iron rod passes through two mutually perpendicular nuts in a way impossible in a real scene (http://yyyggg1398.blog.163.com/blog/static/102113077201041610523293/). [file 1471-2202-15-50-S1.pdf]

Attached picture 2

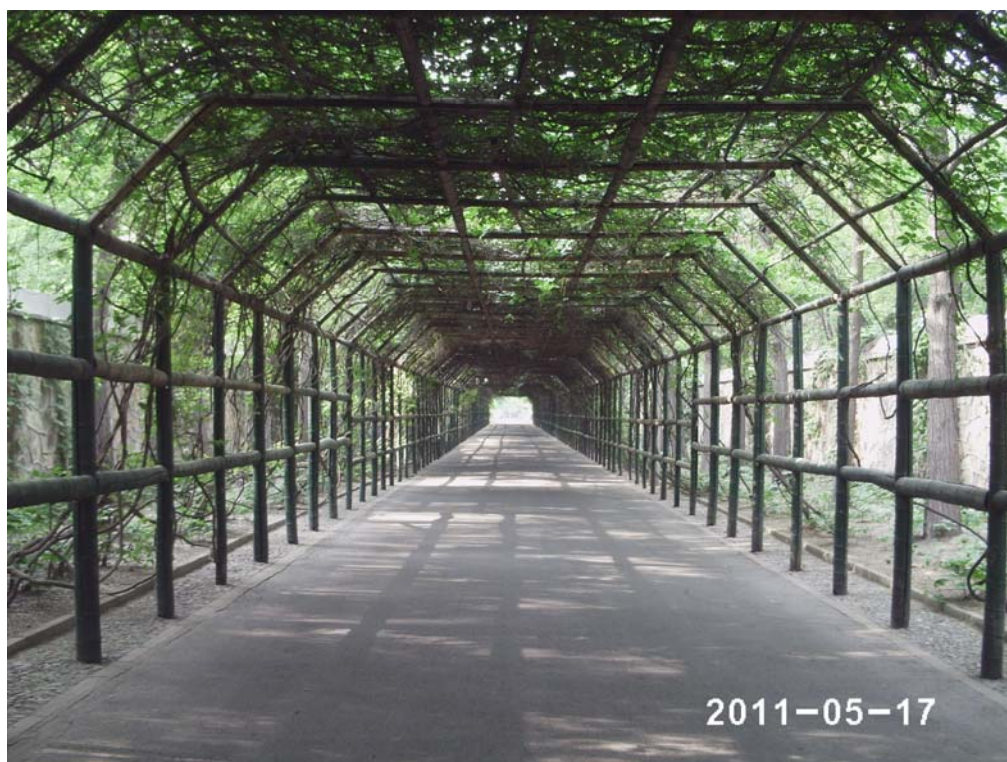

Supplement: Additional file 2 — Visual depth perception in an image of a truss structure. [file 1471-2202-15-50-S2.pdf]

Attached picture 3

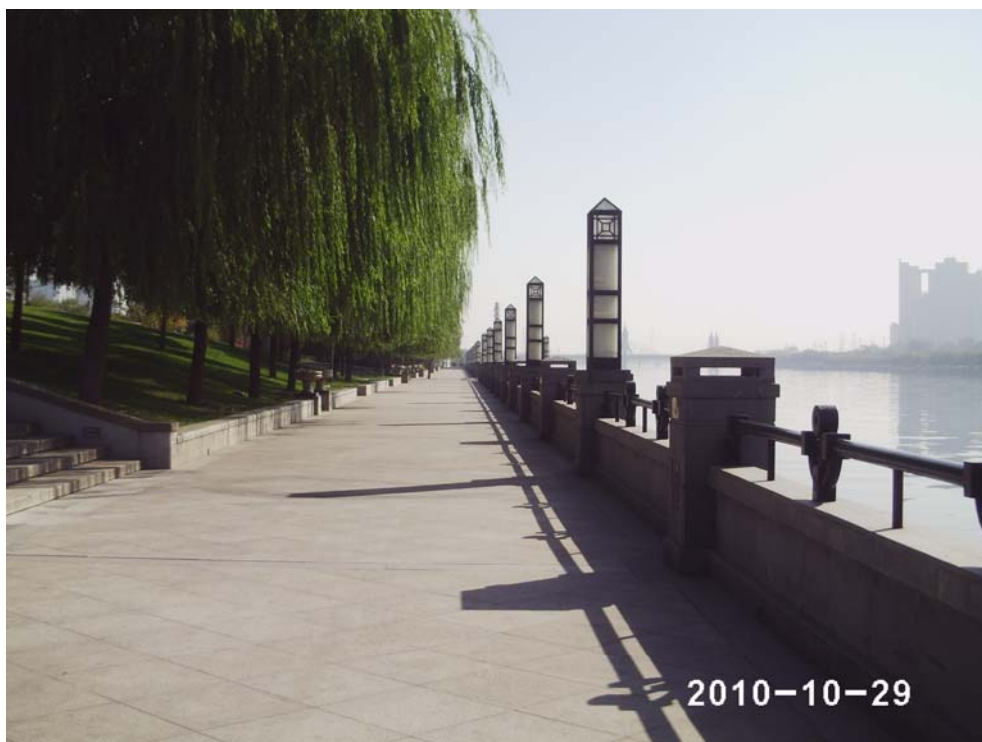

Supplement: Additional file 3 — Visual depth perception in a landscape image. [file 1471-2202-15-50-S3.pdf]

Attached picture 4

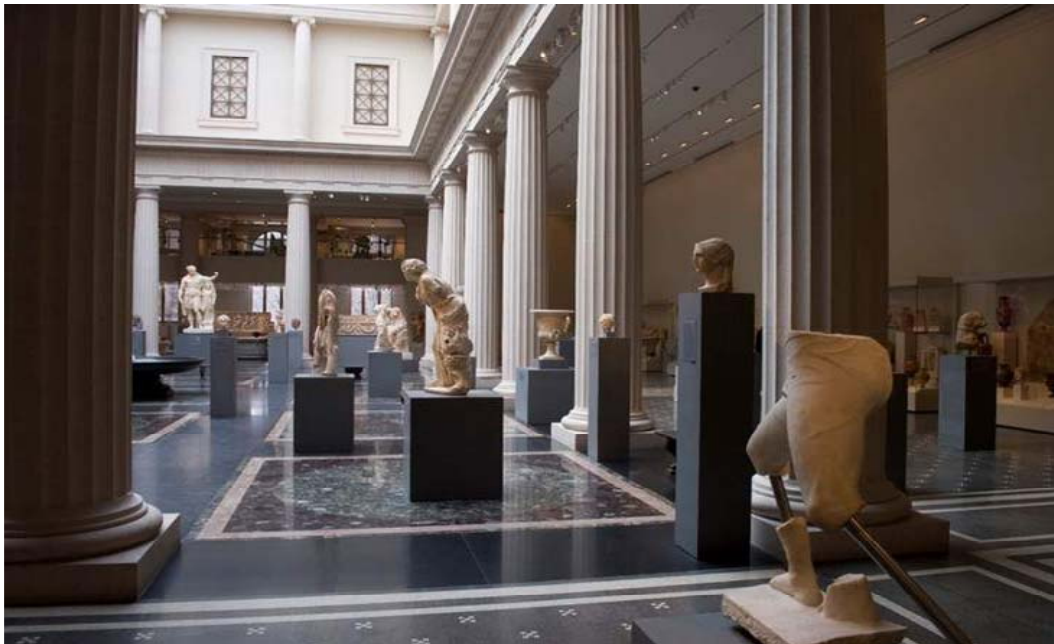

Supplement: Additional file 4 — Three-dimensional scene with stereoscopic visual perception indicating a range of depth at the Metropolitan Museum of Art, New York. [file 1471-2202-15-50-S4.pdf]

Attached picture 5

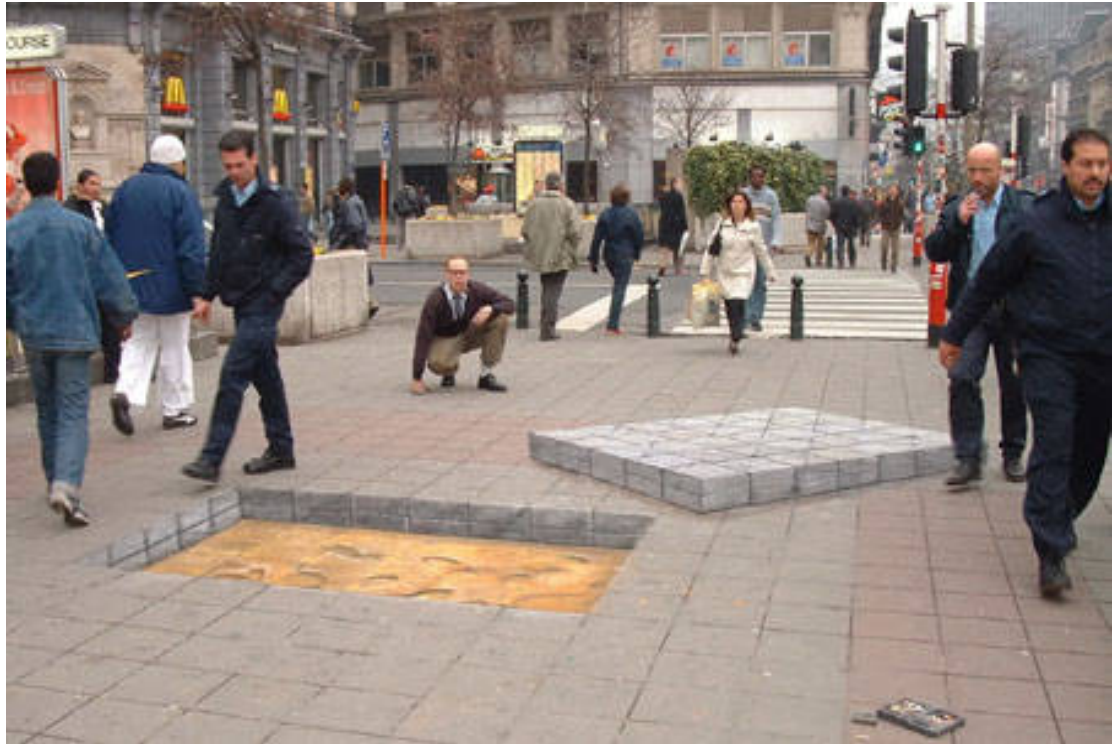

Supplement: Additional file 5 — Vivid effect of three-dimensional perception in a picture painted on the pavement [[9]] (http://yyyggg1398.blog.163.com/blog/static/1021130772010416104031212). [file 1471-2202-15-50-S5.pdf]
